# Income disparities between adult childhood cancer survivors and their peers—A register‐based cohort study from the SALiCCS research programme

**DOI:** 10.1002/cam4.6218

**Published:** 2023-06-12

**Authors:** Anniina Kyrönlahti, Friederike Erdmann, Maria Feychting, Line Elmerdahl Frederiksen, Elli Hirvonen, Liisa Maria Korhonen, Anja Krøyer, Luzius Mader, Nea Malila, Hanna Mogensen, Camilla Pedersen, Mats Talbäck, Mervi Taskinen, Jeanette Falck Winther, Laura Madanat‐Harjuoja, Janne Pitkäniemi

**Affiliations:** ^1^ Finnish Cancer Registry Helsinki Finland; ^2^ Children and Adolescents Helsinki University Hospital and University of Helsinki Helsinki Finland; ^3^ Childhood Cancer Research Group Danish Cancer Society Research Center Copenhagen Denmark; ^4^ Division of Childhood Cancer Epidemiology Institute of Medical Biostatistics, Epidemiology, and Informatics (IMBEI), University Medical Center of the Johannes Gutenberg University Mainz Germany; ^5^ Department of Prevention and Evaluation Leibniz Institute for Prevention Research and Epidemiology – BIPS Bremen Germany; ^6^ Unit of Epidemiology Institute of Environmental Medicine, Karolinska Institutet Stockholm Sweden; ^7^ Childhood Cancer Research Group Institute of Social and Preventive Medicine, University of Bern Bern Switzerland; ^8^ Cancer Registry Bern‐Solothurn University of Bern Bern Switzerland; ^9^ Division of Pediatric Hematology, Oncology, and Stem Cell Transplantation Helsinki University Hospital Helsinki Finland; ^10^ Dana Farber/Boston Children's Cancer and Blood Disorders Clinic Boston Massachusetts USA; ^11^ Health Sciences Unit, Faculty of Social Sciences Tampere University Tampere Finland; ^12^ Department of Public Health, Faculty of Medicine University of Helsinki Helsinki Finland

**Keywords:** adolescent, cancer survivors, child, cohort study, income, Nordic countries, social inequality

## Abstract

**Background:**

Childhood cancer survivors face various adverse consequences. This Nordic register‐based cohort study aimed to assess whether survivors of childhood cancer are more likely to have low income than their peers.

**Methods:**

We identified 17,392 childhood cancer survivors diagnosed at ages 0 to 19 between 1971 and 2009 with 83,221 age‐, sex‐, and country‐matched population comparisons. Annual disposable income at ages 20 to 50 years was retrieved from statistical offices (for 1990–2017) and categorized into low income and middle/high income. The number of transitions between income categories were assessed using binomial regression analyses.

**Results:**

The prevalence of annual low income among childhood cancer survivors was 18.1% and 15.6% among population comparisons (risk ratio [RR] 1.17; 95% confidence interval [CI] 1.16–1.18). Compared to population comparisons, childhood cancer survivors were 10% (95% CI 8%–11%) less likely to transition from low to middle/high income and 12% (10%–15%) more likely to transition from middle/high to low income during follow‐up. Among those initially in the low income category, survivors were 7% (95% CI 3%–11%) more likely to remain in the low income category. If the initial category was middle/high income, childhood cancer survivors were 10% (95% CI 8%–11%) less likely to remain in the middle/high income and 45% (37%–53%) more likely to transition to the low income category permanently.

**Conclusions:**

Childhood cancer survivors are at higher risk for low income in adulthood than their peers. These disparities might be reduced by continued career counseling along with support in managing within the social security system.

## 1 INTRODUCTION

Childhood cancer survival has remarkably improved over the past decades with overall 5‐year survival in most European countries exceeding 80% already during the period 2005–2007, with however substantial variations in survival across cancer types.^1^ Due to the advances in diagnostics and treatment modalities, there is a growing number of childhood cancer survivors reaching adulthood.^2^

Childhood cancer survivors are at risk of a wide spectrum of late effects including somatic and psychiatric conditions.^3^, ^4^ These are likely to contribute to the socioeconomic challenges that childhood cancer survivors have been shown to face later in life.^5^, ^6^ Previous studies have shown that having cancer in childhood might affect educational achievements, employment, social life, or income.^5^, ^6^ Low income has shown to be associated with health‐related behaviors and various adverse health outcomes, such as higher blood pressure and unfavorable cholesterol profile.^7^

A vast majority of previous studies have measured income data at a single time point and studies from North America^8^ and Europe^9^ have overall found income levels to be lower in childhood cancer survivors compared to siblings/population comparisons. Studies from the Nordic countries have found lower income among childhood cancer survivors in most cancer types in Finland,^10^ but only among CNS tumor survivors in Norway^11^ and Sweden.^12^ To our knowledge, only one previous study has investigated income in childhood cancer survivors using longitudinal income data with a possibility to evaluate changes in income over time. This Canadian registry‐based study, using income data between 1982 and 2010, found that young cancer survivors earned significantly less than the general population over the entire follow‐up period and long‐term income was particularly pronounced among survivors treated with radiation.^13^ However, due to cultural and societal differences along with differences in health care and welfare systems the situation in North America may not be comparable to the one in Nordic countries.

By evaluating annual income trajectories using the longitudinal income data, we sought to determine if Nordic childhood cancer survivors are more likely to have low income compared to their peers in the long term. We approached this by evaluating differences in annual transitions from low income to middle/high income and vice versa, between childhood cancer survivors and population comparisons. As a secondary objective we aimed to identify childhood cancer survivors at particular risk for low income. The novelty of our study is the three‐country wide longitudinal high quality annual Nordic register‐based data which allowed us to explore income trends over time in childhood cancer survivors from Denmark, Finland, and Sweden.

## 2 METHODS

### 2.1 Study design, study population, and data sources

This study is based on register data from the Nordic research programme Socioeconomic consequences in Adult Life after Childhood Cancer in Scandinavia (SALiCCS, www.saliccs.org).^14^ The Nordic population‐based registries represent a high quality standard in terms of completeness and accuracy of the comprehensive annual health, socio‐demographic, and socioeconomic data.^15^ Unique personal identification numbers assigned to all residents in the Nordic countries allowed accurate linkage across the registries.

Our study cohort included childhood cancer survivors diagnosed with their first cancer before the age of 20 years. Survivors were identified from the national cancer registries in Denmark, Finland, and Sweden from 1971 to 2009 (in Denmark to 2008) (Figure S1). For each survivor, five age‐ and sex‐matched population comparisons were identified from the national population registries in each country. Childhood cancer survivors who had emigrated before the start of follow‐up (*n* = 113) and survivors with no population comparisons (*n* = 1) were excluded (Figure S1).

Follow‐up began at the age of 20 years or the time‐point from which information on disposable income was available from the registries (in Denmark and Sweden from 1990, in Finland from 1995), whichever occurred latest (Figure S1). If information on income was missing for any given year, the individual with missing income information was excluded for income assessment during that calendar year. Overall, under 0.4% of the income observations were missing. Missing data were not related to case‐comparison status. Follow‐up ended at death, emigration, the age of 50 years, or at the end of follow‐up (August 11, 2017 in Denmark, December 31, 2014 in Finland, and December 31, 2015 in Sweden), whichever occurred first. The matched comparisons were censored when follow‐up ended for the respective survivor. At the beginning of follow‐up, all childhood cancer survivors had at least one population comparison and the majority (*n* = 13,908, 80%) had all five.

Sociodemographic information was retrieved from the national statistical offices. Statistics Denmark,^16^ Statistics Finland,^17^, ^18^, ^19^ and Statistics Sweden^20^ provided annual information on income, studying, and education.

### 2.2 Outcomes

Using individual annual disposable income, we defined two income categories: the low income and the middle/high income. The cutoff limit for low income was based on the at‐risk‐of‐poverty threshold as defined by Eurostat, in which the threshold is set at 60% of the national median disposable income.^21^ The threshold in this study was calculated annually using the disposable income of population comparisons as a reference for each country separately and applied to childhood cancer survivors by age and year of reference. Disposable income in all three countries comprised salaries and allowances including unemployment benefits and deducting taxes paid.^17^, ^20^, ^22^

### 2.3 Covariates

Information on enrollment in any educational program during the calendar year was obtained annually from the national statistical offices for the entire follow‐up period and two categories were defined: studying and not studying. Based on the annual information, follow‐up was constructed and analyzed for those studying and not studying separately. Parental education the year before cancer diagnosis was retrieved from the registries and highest educational level of either parent was used as an explanatory variable.

Calendar period during follow‐up was divided into six categories: 1990–1994, 1995–1999, 2000–2004, 2005–2009, 2010–2014, and 2015–2017. Attained age during follow‐up was divided into six categories: 20–24 years, 25–29 years, 30–34 years, 35–39 years, 40–44 years, and 45–49 years.

For childhood cancer survivors, age at cancer diagnosis was divided into four categories: 0–4 years, 5–9 years, 10–15 years, and 16–19 years. For population comparisons, the index date (i.e., date of start of follow‐up) was defined as the date of the cancer diagnosis of their matched survivor. Diagnostic period (calendar period of index date for population comparisons) was divided into four categories: 1971–1979, 1980–1989, 1990–1999, and 2000–2009. Cancer diagnoses were categorized according to International Classification of Childhood Cancer (ICCC)^23^ into the 12 main diagnostic groups for analyses.

### 2.4 Statistical methods

First, we calculated the prevalence of low and middle/high income for childhood cancer survivors and population comparisons during the entire follow‐up period and by the age of 35. Analyses by the age of 35 were stratified by diagnostic group. The cutoff age was chosen as 35 years based on the assumption that if a person was to rise up from the low income trap, they would have done so by that age. Progression of income was described by transitions between two consecutive years, starting from the initial category of income, which was defined at the start of follow‐up as low or middle/high income. We compared annual transitions from low income to middle/high income and from middle/high income to low income between childhood cancer survivors and population comparisons. Each individual contributed one observation per year to the aforementioned analyses. The analyses including the overall number of transitions from low income to middle/high income, and vice versa were stratified by sex, attained age during follow‐up, calendar period of follow‐up, student status, age at diagnosis, diagnostic group, diagnostic period, and country. For pairwise differences of the prevalence in low and middle/high income categories and transitions between the two income categories between survivors and comparisons, we estimated risk ratios (RRs) with 95% confidence intervals (CIs) using binomial regression modeling. All relative risks were obtained from standard generalized linear model with log‐link function and likelihood based statistical inference. Analyses were adjusted for attained age during follow‐up, calendar period of follow‐up, sex, and country, when applicable. Additional adjustment included highest parental education. Statistical method applied in our analysis has been published by Whalen, et al.^24^

To explore if childhood cancer survivors were more likely to permanently remain in the low income category than population comparisons, we studied trajectories of low and middle/high income based on annual individual transitions and initial category of income. We identified differences in variation dynamics between cancer survivors and their comparisons by estimating four summary statistics by initial category.^24^ If the initial category was low income, the categories were as follows: (1) remaining in low income category (no transitions), (2) permanent transition to middle/high income (one transition), (3) transition to middle/high income and permanently back to low income (two transitions), or (4) multiple transitions. If the initial category was middle/high income, the categories were as follows: (1) remaining in the middle/high income category, (2) permanent transition to low income, (3) transition to low income and permanently back to middle/high income, or (4) multiple transitions. Differences of individual transitions were compared between survivors and population comparisons and RRs with 95% CIs were calculated using binomial regression modeling adjusted for sex, country, and additional analyses including the highest parental education. All of the aforementioned trajectory analyses were stratified by diagnostic group with four main groups: leukemias, lymphomas, CNS tumors, and other solid tumors (ICCC‐3 groups IV‐XII combined) and the stratified analyses were corrected for multiple comparisons using the Benjamini & Hochberg procedure.^25^

We performed sensitivity analyses using only 5‐year survivors of childhood cancer to control for the possible effect of ongoing cancer treatment. An analysis with follow‐up starting at the age of 30 years and ending at the age of 40 years was also performed to include only individuals who most likely had attained a stable socioeconomic position.

Statistical analyses were conducted with R software version 4.1.0 (R project for Statistical Analysis) and packages data.table (version 1.14.0) and Epi (version 2.44).

## 3 RESULTS

### 3.1 Characteristics of the study population

A total of 17,392 childhood cancer survivors and 83,221 matched population comparisons were included in the study (Table 1). The median follow‐up time was 18 years for survivors and 17 years for population comparisons (interquartile range [IQR] 10). Median age at the end of follow‐up was 33 years for survivors and 32 years for comparisons (IQR 16).

**TABLE 1:** Characteristics of childhood cancer survivors (diagnosed with cancer at the age of 0 to 19 years between 1971 and 2009) and their population comparisons (matched by birth year, sex, and country).

	Survivors	Population comparisons
	*N* (%)	*N* (%)
Total	17,392 (100)	83,221 (100)
Sex		
Male	9211 (53.0)	44,211 (53.1)
Female	8181 (47.0)	39,010 (46.9)
Country		
Denmark	4721 (27.1)	21,745 (26.1)
Finland	4565 (26.2)	22,345 (26.9)
Sweden	8106 (46.6)	39,131 (47.0)
Diagnostic group (by ICCC)		
Leukemias	3423 (19.7)	NA
Lymphomas	2805 (16.1)	NA
CNS tumors^a^	4255 (24.5)	NA
Neuroblastomas	344 (2.0)	NA
Retinoblastomas	343 (2.0)	NA
Renal tumors	698 (4.0)	NA
Hepatic tumors	85 (0.5)	NA
Bone tumors	712 (4.1)	NA
Soft tissue sarcomas	878 (5.0)	NA
Germ‐cell tumors	1281 (7.4)	NA
Carcinomas	2157 (12.4)	NA
Other and unspecified neoplasms	411 (2.4)	NA
Age at diagnosis/age at index date^b^		
0–4 years	3972 (22.8)	18,628 (22.3)
5–9 years	2863 (16.5)	13,673 (16.4)
10–15 years	4824 (27.7)	23,316 (28.0)
16–19 years	5733 (32.0)	27,754 (33.3)
Diagnostic period/calendar period of index date^b^		
1971–1979	2804 (16.1)	13,071 (15.7)
1980–1989	4833 (27.8)	23,154 (27.8)
1990–1999	5988 (34.4)	28,845 (34.6)
2000–2009	3767 (21.7)	18,272 (21.9)

*Note*: From 1971 to 2008 in Denmark.

Abbreviation: ICCC, International Classification of Childhood Cancer.

^a^ Central nervous system tumors.

^b^ For population comparisons.

Observations in low and middle/high income categories showed that among survivors, 18.1% of the follow‐up time was spent in the low income category versus 15.6% among population comparisons (RR 1.17 [95% CI 1.16–1.18]), respectively (Table 2). By the age of 35 years, the prevalence in low income was 18.6% among survivors and 16.6% among population comparisons (RR 1.17 [95% CI 1.16–1.18]). Survivors of any childhood cancer type except survivors of other and unspecified neoplasms were more likely to spend more time in the low income category by the age of 35 years compared to population comparisons (Table 2).

**TABLE 2:** The prevalence of low and middle/high income and adjusted RRs with 95% CIs for low income in childhood cancer survivors compared to population comparisons (matched by birth year, sex, and country).

	Survivors		Population comparisons			
	Low income	Middle/high income	Low income	Middle/high income		
	*N* (%)	*N* (%)	*N* (%)	*N* (%)	Adjusted^a^ RR (95% CI)	Adjusted^b^ RR (95% CI)
During the entire study period	41,321 (18.1)	187,055 (81.9)	164,812 (15.6)	890,253 (84.4)	1.17 (1.16–1.18)	1.15 (1.14–1.16)
By the age of 35	31,583 (18.6)	138,056 (81.4)	131,973 (16.6)	661,636 (83.4)	1.17 (1.16–1.18)	1.15 (1.14–1.16)
By the age of 35 by diagnostic group						
Leukemias	7681 (19.8)	31,147 (80.2)	29,644 (16.6)	148,862 (83.4)	1.20 (1.17–1.23)	1.17 (1.14–1.20)
Lymphomas	6632 (17.5)	31,352 (82.5)	28,298 (15.9)	149,292 (84.1)	1.10 (1.08–1.13)	1.09 (1.06–1.12)
CNS tumors^c^	11,391 (20.5)	44,212 (79.5)	39,376 (15.3)	217,940 (84.7)	1.35 (1.33–1.38)	1.32 (1.29–1.34)
Neuroblastomas	740 (21.1)	2774 (78.9)	2693 (16.9)	13,222 (83.1)	1.26 (1.17–1.35)	1.23 (1.12–1.34)
Retinoblastomas	823 (18.3)	3674 (81.7)	3165 (15.9)	16,708 (84.1)	1.18 (1.10–1.26)	1.19 (1.10–1.29)
Renal tumors	1463 (16.7)	7323 (83.3)	6136 (15.6)	33,193 (84.4)	1.08 (1.03–1.14)	1.07 (1.00–1.13)
Hepatic tumors	147 (22.2)	514 (77.8)	628 (19.9)	2533 (80.1)	1.15 (0.99–1.34)	NA^d^
Bone tumors	1552 (16.9)	7615 (83.1)	6654 (15.5)	36,290 (84.5)	1.10 (1.04–1.15)	1.07 (1.01–1.13)
Soft tissue sarcomas	1920 (17.6)	9016 (82.4)	8154 (16.1)	42,451 (83.9)	1.10 (1.05–1.15)	1.10 (1.05–1.16)
Germ‐cell tumors	2982 (15.9)	15,742 (84.1)	13,162 (15.3)	72,666 (84.7)	1.04 (1.01–1.08)	1.06 (1.02–1.10)
Carcinomas	5051 (15.2)	28,255 (84.8)	22,525 (14.6)	131,674 (85.4)	1.04 (1.02–1.08)	1.03 (1.00–1.07)
Other and unspecified neoplasms	938 (14.7)	5431 (85.3)	4377 (14.7)	25,422 (85.3)	1.01 (0.95–1.08)	0.98 (0.91–1.05)

*Note*: Prevalence–Each individual contributed one observation per year to the analyses. Income–Annual disposable income at ages 20 to 50 years was retrieved between 1990 and 2017 for Denmark, 1990 and 2015 for Sweden, and 1995 and 2014 for Finland, and dichotomized to low income and middle/high income based on the at‐risk‐of‐poverty threshold defined by Eurostat. Survivors–Diagnosed with cancer at the age of 0 to 19 years from 1971 to 2009 for Finland and Sweden, and from 1971 to 2008 for Denmark.

Abbreviation: ICCC, International Classification of Childhood Cancer.

^a^ Adjusted for attained age during follow‐up, calendar period, sex, and country.

^b^ Adjusted for attained age during follow‐up, calendar period, sex, country, and highest parental education.

^c^ Central nervous system tumors.

^d^ Could not bet counted due to the low number of observations.

### 3.2 Transitions between low and middle/high income

**FIGURE 1:**
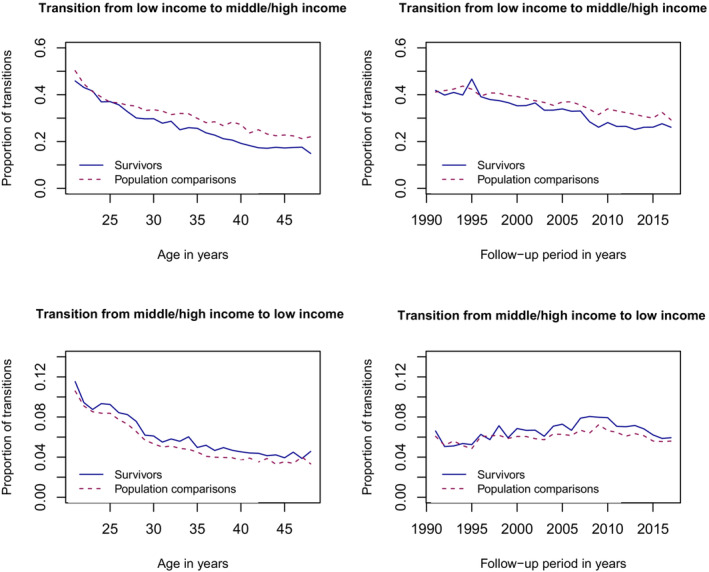
Proportion of transitions from low income to middle/high income and from middle/high income to low income, by age and survivorship, and follow‐up period and survivorship among childhood cancer survivors (diagnosed with cancer at the age of 0 to 19 years from 1971 to 2009 in Finland and Sweden, and from 1971 to 2008 in Denmark) and their population comparisons (matched by birth year, sex, and country).

Compared to population comparisons, childhood cancer survivors had overall less transitions from the low to the middle/high income category (RR 0.90 [95% CI 0.89–0.92]) (Table 3) and more transitions from the middle/high to the low income category (RR 1.12 [1.10–1.14]) (Table 4) during the follow‐up period. This pattern was evident during all calendar periods and in all age groups with the differences even increasing by age (Figure 1, Tables 3 and 4).

**TABLE 3:** Characteristics and adjusted RRs with 95% CIs for number of transitions from low income to middle/high income for childhood cancer survivors compared to population comparisons (matched by birth year, sex, and country).

	Survivors		Population comparisons			
	Number of individuals remaining in low income (%)^a^	Number of transitions to middle/high income (%)^a^	Number of individuals remaining in low income (%)^a^	Number of transitions to middle/high income (%)^a^	Adjusted^b^ RR (95% CI)	Adjusted^c^ RR (95% CI)
Total	25,598 (69.0)	11,503 (31.0)	96,569 (64.6)	52,819 (35.4)	0.90 (0.89–0.92)	0.92 (0.90–0.94)
Sex						
Male	13,461 (69.9)	5803 (30.1)	53,502 (66.0)	27,545 (34.0)	0.92 (0.90–0.94)	0.94 (0.92–0.96)
Female	12,137 (68.0)	5700 (32.0)	43,067 (63.0)	25,274 (37.0)	0.95 (0.93–0.97)	0.96 (0.94–0.98)
Follow‐up period						
1990–1994	924 (59.5)	630 (40.5)	4353 (57.6)	3200 (42.4)	0.96 (0.90–1.03)	0.95 (0.89–1.02)
1995–1999	2551 (61.3)	1614 (38.8)	11,304 (59.6)	7662 (40.4)	0.97 (0.93–1.01)	0.97 (0.93–1.02)
2000–2004	4436 (65.3)	2358 (34.7)	17,854 (62.7)	10,624 (37.3)	0.95 (0.91–0.98)	0.97 (0.93–1.00)
2005–2009	6684 (69.4)	2946 (30.6)	24,991 (65.2)	13,328 (34.8)	0.90 (0.87–0.93)	0.93 (0.89–0.96)
2010–2014	9837 (73.6)	3532 (26.4)	33,666 (67.7)	16,066 (32.3)	0.84 (0.82–0.87)	0.86 (0.83–0.89)
2015–2017	1166 (73.4)	423 (26.6)	4401 (69.4)	1939 (30.6)	0.90 (0.82–0.98)	0.89 (0.82–0.97)
Attained age during follow‐up						
20–24 years	5806 (57.7)	4257 (42.3)	25,407 (55.6)	20,256 (44.4)	0.96 (0.93–0.98)	0.96 (0.93–0.98)
25–29 years	6522 (66.8)	3246 (33.2)	26,641 (64.4)	14,704 (35.6)	0.94 (0.91–0.97)	0.95 (0.92–0.98)
30–34 years	4938 (72.4)	1882 (27.6)	17,646 (67.5)	8487 (32.5)	0.86 (0.82–0.89)	0.88 (0.84–0.92)
35–39 years	3895 (77.1)	1160 (23.0)	12,675 (71.7)	5016 (28.4)	0.81 (0.76–0.85)	0.82 (0.77–0.87)
40–44 years	2822 (82.0)	618 (18.0)	8788 (75.5)	2853 (24.5)	0.74 (0.69–0.80)	0.74 (0.68–0.81)
45–49 years	1615 (82.6)	340 (17.4)	5412 (78.3)	1503 (21.7)	0.81 (0.73–0.90)	0.86 (0.77–0.97)
Age at diagnosis						
0–4 years	5526 (69.4)	2437 (30.6)	18,224 (62.7)	10,833 (37.3)	0.87 (0.84–0.90)	0.88 (0.85–0.92)
5–9 years	4289 (70.1)	1826 (29.9)	15,158 (63.7)	8647 (36.3)	0.85 (0.82–0.89)	0.89 (0.84–0.92)
10–15 years	7495 (69.8)	3242 (30.2)	27,415 (64.9)	14,853 (35.1)	0.89 (0.86–0.92)	0.90 (0.88–0.93)
16–19 years	8288 (67.5)	3998 (32.5)	34,999 (66)	18,024 (34)	0.97 (0.95–1.00)	0.97 (0.95–1.00)
Diagnostic period						
1971–1979	5746 (75.3)	1884 (24.7)	18,764 (68.3)	8722 (31.7)	0.81 (0.78–0.85)	0.83 (0.79–0.87)
1980–1989	9676 (70.2)	4116 (29.8)	34,937 (65.6)	18,297 (34.4)	0.90 (0.87–0.92)	0.93 (0.90–0.97)
1990–1999	7315 (65.7)	3815 (34.3)	29,643 (62.5)	17,753 (37.5)	0.93 (0.90–0.96)	0.93 (0.90–0.96)
2000–2009	2861 (62.9)	1688 (37.1)	12,452 (62.2)	7585 (37.9)	0.99 (0.95–1.03)	0.98 (0.94–1.03)
Diagnostic group (by ICCC)						
Leukemias	4718 (68.7)	2146 (31.3)	16,850 (63.8)	9544 (36.2)	0.88 (0.85–0.92)	0.90 (0.86–0.94)
Lymphomas	3970 (66.2)	2029 (33.8)	16,613 (65.1)	8918 (34.9)	0.98 (0.94–1.01)	0.99 (0.95–1.03)
CNS tumors^d^	7431 (73.2)	2714 (26.8)	22,583 (64.0)	12,702 (36.0)	0.81 (0.78–0.83)	0.82 (0.79–0.85)
Neuroblastomas	454 (70.5)	190 (29.5)	1479 (62.4)	890 (37.6)	0.81 (0.71–0.92)	0.87 (0.76–1.01)
Retinoblastomas	510 (68.6)	233 (31.4)	1773 (61.4)	1113 (38.6)	0.86 (0.77–0.96)	0.84 (0.74–0.96)
Renal tumors	835 (63.3)	484 (36.7)	3465 (62.9)	2043 (37.1)	1.00 (0.93–1.08)	1.00 (0.92–1.09)
Hepatic tumors	83 (70.3)	35 (29.7)	334 (63.5)	192 (36.5)	0.82 (0.61–1.11)	0.80 (0.57–1.13)
Bone tumors	959 (69.1)	428 (30.9)	3904 (66.2)	1993 (33.8)	0.94 (0.86–1.02)	0.98 (0.90–1.07)
Soft tissue sarcomas	1186 (69.6)	517 (30.4)	4759 (65.4)	2513 (34.6)	0.90 (0.83–0.97)	0.93 (0.86–1.01)
Germ‐cell tumors	1828 (68.0)	862 (32.0)	7843 (65.6)	4120 (34.4)	0.94 (0.89–1.00)	0.94 (0.88–1.00)
Carcinomas	3045 (65.6)	1595 (34.4)	13,537 (65.9)	6994 (34.1)	1.01 (0.97–1.06)	1.02 (0.98–1.07)
Other and unspecified neoplasms	579 (68.2)	270 (31.8)	2656 (66.5)	1335 (33.5)	0.96 (0.87–1.07)	1.03 (0.92–1.15)
Studying						
Total	6255 (71.4)	2512 (28.7)	26,832 (69.7)	11,679 (30.3)	0.94 (0.91–0.98)	0.96 (0.92–0.99)
Male	3156 (72.8)	1182 (27.3)	14,051 (70.9)	5757 (29.1)	0.96 (0.91–1.01)	0.96 (0.90–1.01)
Female	3099 (70.0)	1330 (30.0)	12,781 (68.3)	5922 (31.7)	0.93 (0.89–0.98)	0.95 (0.91–1.00)
Not studying						
Total	19,343 (68.3)	8991 (31.7)	69,737 (62.9)	41,140 (37.1)	0.89 (0.88–0.91)	0.91 (0.89–0.93)
Male	10,305 (69.0)	4621 (31.0)	39,451 (64.4)	21,788 (35.6)	0.91 (0.89–0.93)	0.93 (0.91–0.96)
Female	9038 (67.4)	4370 (32.6)	30,286 (61.0)	19,352 (39.0)	0.87 (0.85–0.89)	0.88 (0.85–0.90)

*Note*: Transitions–Each individual contributed one observation per year to the analyses. Income–Annual disposable income at ages 20 to 50 years was retrieved between 1990 and 2017 for Denmark, 1990 and 2015 for Sweden, and 1995 and 2014 for Finland, and dichotomized to low income and middle/high income based on the at‐risk‐of‐poverty threshold defined by Eurostat. Survivors–Diagnosed with cancer at the age of 0 to 19 years from 1971 to 2009 for Finland and Sweden, and from 1971 to 2008 for Denmark.

^a^ Total number of yearly observations during the entire follow‐up time.

^b^ Adjusted for attained age during follow‐up, calendar period, sex and country, when applicable.

^c^ Adjusted for attained age during follow‐up, calendar period, sex, country, and highest parental education, when applicable.

^d^ Central nervous system tumors.

**TABLE 4:** Characteristics and adjusted RRs with 95% CIs for number of transitions from middle/high income to low income for childhood cancer survivors compared to population comparisons (matched by birth year, sex, and country).

	Survivors		Population comparisons			
	Number of individuals remaining in middle/high income (%)^a^	Number of transitions to low income (%)^a^	Number of individuals remaining in middle/high income (%)^a^	Number of transitions to low income (%)^a^	Adjusted^b^ RR (95% CI)	Adjusted^c^ RR (95% CI)
Total	162,117 (93.1)	11,940 (6.9)	772,611 (93.8)	50,776 (6.2)	1.12 (1.10–1.15)	1.12 (1.10–1.14)
Sex						
Male	86,245 (93.5)	5998 (6.5)	409,110 (94.2)	25,173 (5.8)	1.13 (1.10–1.16)	1.13 (1.09–1.16)
Female	75,872 (92.7)	5942 (7.3)	363,501 (93.4)	25,603 (6.6)	1.12 (1.10–1.15)	1.11 (1.08–1.15)
Follow‐up period						
1990–1994	10,658 (94.5)	619 (5.5)	50,215 (94.5)	2905 (5.5)	1.01 (0.93–1.10)	0.98 (0.90–1.07)
1995–1999	24,166 (93.9)	1578 (6.1)	114,024 (94.1)	7126 (5.9)	1.05 (1.00–1.11)	1.06 (1.00–1.13)
2000–2004	33,413 (93.3)	2389 (6.7)	159,281 (94.0)	10,168 (6.0)	1.12 (1.08–1.17)	1.11 (1.05–1.16)
2005–2009	40,189 (92.4)	3301 (7.6)	193,451 (93.4)	13,582 (6.6)	1.17 (1.12–1.21)	1.17 (1.12–1.22)
2010–2014	45,818 (92.8)	3550 (7.2)	222,481 (93.7)	15,033 (6.3)	1.15 (1.11–1.19)	1.15 (1.11–1.19)
2015–2017	7873 (93.0)	503 (6.0)	33,159 (94.4)	1962 (5.6)	1.09 (0.99–1.20)	1.09 (0.99–1.20)
Attained age during follow‐up						
20–24 years	35,571 (90.2)	3846 (9.8)	174,230 (90.9)	17,507 (9.1)	1.07 (1.04–1.11)	1.07 (1.03–1.11)
25–29 years	40,321 (92.0)	3501 (8.0)	193,370 (92.8)	14,965 (7.2)	1.12 (1.08–1.16)	1.09 (1.05–1.14)
30–34 years	33,565 (94.2)	2068 (5.8)	158,608 (95.0)	8399 (5.0)	1.17 (1.12–1.22)	1.16 (1.10–1.22)
35–39 years	25,612 (95.1)	1319 (4.9)	120,717 (95.9)	5177 (4.1)	1.20 (1.13–1.28)	1.21 (1.14–1.29)
40–44 years	17,263 (95.7)	786 (4.4)	80,662 (96.3)	3073 (3.7)	1.19 (1.10–1.29)	1.23 (1.13–1.34)
45–49 years	9785 (95.9)	420 (4.1)	45,024 (96.5)	1655 (3.6)	1.16 (1.05–1.29)	1.28 (1.14–1.44)
Age at diagnosis						
0–4 years	28,436 (91.9)	2501 (8.1)	134,700 (93.1)	10,043 (6.9)	1.17 (1.12–1.22)	1.14 (1.08–1.19)
5–9 years	24,733 (93.0)	1864 (7.0)	116,136 (93.4)	8234 (6.6)	1.07 (1.02–1.12)	1.07 (1.01–1.12)
10–15 years	47,028 (93.3)	3382 (6.7)	225,791 (94.0)	14,425 (6.0)	1.13 (1.09–1.17)	1.13 (1.09–1.18)
16–19 years	61,920 (93.7)	4193 (6.3)	290,597 (94.3)	17,684 (5.7)	1.12 (1.08–1.15)	1.12 (1.08–1.16)
Diagnostic period						
1971–1979	40,447 (95.0)	2138 (5.0)	186,430 (95.5)	8839 (4.5)	1.12 (1.07–1.17)	1.12 (1.06–1.18)
1980–1989	63,040 (93.6)	4347 (6.5)	300,262 (94.4)	17,818 (5.6)	1.16 (1.12–1.20)	1.15 (1.11–1.20)
1990–1999	43,949 (91.9)	3863 (8.1)	210,073 (92.7)	16,638 (7.3)	1.11 (1.08–1.15)	1.11 (1.07–1.15)
2000–2009	14,681 (90.2)	1592 (9.8)	70,459 (90.9)	7091 (9.1)	1.08 (1.02–1.13)	1.08 (1.02–1.14)
Diagnostic group (by ICCC)						
Leukemias	26,436 (92.4)	2168 (7.6)	125,988 (93.3)	9098 (6.7)	1.14 (1.09–1.19)	1.12 (1.07–1.18)
Lymphomas	27,173 (93.1)	2023 (6.9)	128,704 (93.8)	8506 (6.2)	1.13 (1.08–1.18)	1.12 (1.07–1.18)
CNS tumors^d^	38,217 (92.7)	3024 (7.3)	187,754 (93.9)	12,214 (6.1)	1.21 (1.16–1.25)	1.19 (1.14–1.24)
Neuroblastomas	2344 (92.5)	189 (7.5)	10,991 (93.1)	811 (6.9)	1.10 (0.95–1.28)	1.08 (0.90–1.29)
Retinoblastomas	3184 (93.1)	235 (6.9)	14,354 (93.4)	1017 (6.6)	1.06 (0.93–1.22)	1.05 (0.90–1.22)
Renal tumors	6316 (93.2)	462 (6.8)	28,513 (93.8)	1876 (6.2)	1.13 (1.02–1.24)	1.12 (1.00–1.25)
Hepatic tumors	425 (91.8)	38 (8.2)	2043 (92.3)	170 (7.7)	1.11 (0.79–1.55)	1.01 (0.69–1.49)
Bone tumors	6621 (93.6)	452 (6.4)	31,275 (94.0)	1984 (6.0)	1.08 (0.98–1.19)	1.13 (1.02–1.25)
Soft tissue sarcomas	7822 (93.5)	544 (6.5)	36,354 (93.8)	2413 (6.2)	1.05 (0.96–1.15)	1.11 (1.01–1.23)
Germ‐cell tumors	13,856 (93.9)	900 (6.1)	63,391 (94.1)	3970 (5.9)	1.05 (0.98–1.12)	1.07 (0.99–1.15)
Carcinomas	24,899 (93.9)	1617 (6.1)	115,554 (94.3)	6998 (5.7)	1.08 (1.03–1.14)	1.07 (1.01–1.13)
Other and unspecified neoplasms	4824 (94.4)	0,288 (5.6)	22,303 (94.4)	1329 (5.6)	1.01 (0.89–1.14)	1.06 (0.93–1.20)
Studying						
Total	15,550 (81.6)	3515 (18.4)	76,654 (82.4)	16,328 (17.6)	1.01 (0.97–1.04)	1.01 (0.98–1.05)
Male	9116 (79.0)	1806 (21.0)	45,510 (80.1)	8585 (19.9)	1.01 (0.97–1.06)	1.03 (0.98–1.08)
Female	6434 (83.5)	1709 (16.5)	31,144 (84.1)	7743 (15.9)	1.00 (0.96–1.05)	1.00 (0.95–1.04)
Not studying						
Total	146,567 (94.6)	8425 (5.4)	695,957 (95.3)	34,448 (4.7)	1.16 (1.13–1.19)	1.16 (1.13–1.20)
Male	79,811 (94.9)	4289 (5.1)	377,966 (95.6)	17,430 (4.4)	1.16 (1.12–1.20)	1.16 (1.12–1.20)
Female	66,756 (94.2)	4136 (5.8)	317,991 (94.9)	17,018 (5.1)	1.16 (1.13–1.20)	1.16 (1.13–1.20)

*Note*: Transitions–Each individual contributed one observation per year to the analyses. Income–Annual disposable income at ages 20 to 50 years was retrieved between 1990 and 2017 for Denmark, 1990 and 2015 for Sweden, and 1995 and 2014 for Finland, and dichotomized to low income and middle/high income based on the at‐risk‐of‐poverty threshold defined by Eurostat. Survivors–Diagnosed with cancer at the age of 0 to 19 years from 1971 to 2009 for Finland and Sweden, and from 1971 to 2008 for Denmark.

^a^ Total number of yearly observations during the entire follow‐up time.

^b^ Adjusted for attained age during follow‐up, calendar period, sex, and country, when applicable.

^c^ Adjusted for attained age during follow‐up, calendar period, sex, country, and highest parental education, when applicable.

^d^ Central nervous system tumors.

We found that childhood cancer survivors both studying and not studying had less transitions from low income to middle/high income (studying RR 0.94 [95% CI 0.91–0.98]; not studying RR 0.89 [0.88–0.91]) compared to population comparisons (Table 3). There were no differences in the number of transitions from middle/high income to low income between survivors and comparisons who were studying (RR 1.01 [95% CI 1.97–1.04]), but in the group not studying the risk of transitioning to low income was increased among survivors (RR 1.16 [1.13–1.19]) (Table 4).

Survivors of leukemias, CNS tumors, neuroblastomas, retinoblastomas, and soft tissue sarcomas had less transitions from low income to middle/high income compared to population comparisons (Table 3). The difference between survivors and population comparisons was seen in all diagnostic age groups and periods, except in the transitions from low to middle/high income among the group 16 to 19 years of age at diagnosis and in the most recent diagnostic period between 2000 and 2009, where the number of transitions were similar between survivors and comparisons. Survivors of leukemias, lymphomas, CNS tumors, renal tumors, and carcinomas had more transitions from middle/high income to low income compared to population comparisons independent of age or period of diagnosis (Table 4).

At the beginning of follow‐up, about 1/5 of survivors and population comparisons were in the low income category (Table 5). Among those with low income at beginning of follow‐up, a similar proportion of survivors and population comparisons had no transitions and remained in low income for the rest of follow‐up. Conversely, childhood cancer survivors starting from low income were less likely to have a permanent transition to middle/high income (RR 0.93 [95% CI 0.89–0.97]), and more likely to have a permanent transition from middle/high income back to low income (RR 1.18 [1.08–1.30]). Survivors of leukemia were less likely to have a permanent transition from low to middle/high income compared to population comparisons (Table S1).

**TABLE 5:** Number of individuals in different states of income among childhood cancer survivors (diagnosed with cancer at the age of 0 to 19 years from 1971 to 2009) and their population comparisons (matched by birth year, sex, and country) by initial state of income.

	Survivors	Population comparisons		
	N (%)	N (%)	Adjusted^a^ RR (95% CI)	Adjusted^b^ RR (95% CI)
Initial category low income	3508 (21.3)	16,647 (20.9)		
Remaining in low income during the entire follow‐up	461 (13.1)	2236 (13.4)	0.98 (0.89–1.08)	1.00 (0.90–1.11)
Permanent transition to middle/high income	1380 (39.3)	7160 (43.0)	0.93 (0.89–0.97)	0.94 (0.89–0.98)
Transition to middle/high income and permanently back to low income	472 (13.5)	1863 (11.2)	1.18 (1.08–1.30)	1.29 (1.07–1.32)
Multiple transitions	1195 (34.1)	5388 (32.4)	1.04 (0.99–1.09)	1.03 (0.97–1.08)
Initial category middle/high income	12,987 (78.7)	62,920 (79.1)		
Remaining in middle/high income during the entire follow‐up	6826 (52.6)	36,483 (58.0)	0.90 (0.89–0.92)	0.91 (0.89–0.93)
Permanent transition to low income	1508 (11.6)	5054 (8.0)	1.45 (1.37–1.53)	1.42 (1.34–1.51)
Transition to low income and permanently back to middle/high income	2346 (18.1)	11,559 (18.4)	0.99 (0.95–1.03)	0.99 (0.95–1.03)
Multiple transitions	2307 (17.8)	9824 (15.6)	1.14 (1.09–1.19)	1.13 (1.08–1.18)

*Note*: From 1971 to 2008 in Denmark.

^a^ Adjusted for sex, and country.

^b^ Adjusted for sex, country, and highest parental education.

If the initial income category was middle/high income, childhood cancer survivors were less likely to stay in the middle/high income category for the remaining follow‐up (RR 0.90 [95% CI 0.89–0.92]), and more likely to have a permanent transition to low income (RR 1.45 [1.37–1.53]) (Table 5). Survivors of leukemias, lymphomas, CNS tumors, and other solid tumors were less likely to remain in the middle/high income category compared to population comparisons and survivors of leukemias, CNS tumors, and other solid tumors were more likely to have a permanent transition to the low income category compared to population comparisons (Table S1).

Analyses stratified by country revealed similar findings (Tables S2‐S4). The sensitivity analyses including only 5‐year survivors of childhood cancer (Tables S5 and S6) and survivors with follow‐up starting at the age of 30 years and ending at the age of 40 years (Table S7) mainly yielded similar results. In the analyses at ages 30–40 years the initial income category was more likely to be low income among survivors than comparisons (16% vs. 13.7%). Adjusting for parental education showed similar results in all analyses and there was no difference in the results by sex.

## 4 DISCUSSION

We found that childhood cancer survivors had overall higher prevalence of low income compared to population comparisons. Survivors were overall less likely to transition from low income to middle/high income and more likely to transition from middle/high income to low income than population comparisons. Childhood cancer survivors were also more likely to have a permanent shift from middle/high income to low income compared to population comparisons, and it appeared to be less likely for childhood cancer survivors to permanently transition from low to middle/high income. The pattern was evident across different calendar periods and age groups with the difference increasing by age.

We used the at‐risk‐of‐poverty threshold by Eurostat^21^ to explore whether childhood cancer survivors were at risk for possible income deprivation. Our longitudinal data with repeated measurement also enabled us to explore whether the income deprivation was persistent over time. We showed that childhood cancer survivors had a persistently higher risk for low income compared to population comparisons and the difference was even increasing by attained age during follow‐up. This finding is in line with the Canadian longitudinal study, where attained age was negatively associated with income.^13^ Our study, however, differs from the Canadian study where they investigated absolute income differences, not the risk for income deprivation. In previous studies, childhood cancer survivors have been shown to have lower educational qualifications than the general population or siblings^7^ or to be at an increased risk for unemployment,^7^, ^10^ which along with early retirement are probable explanatory factors underlying low income. Inability to work for example, due to disability may contribute to this, as seen in another SALiCCS study that showed childhood cancer survivors having higher odds of health‐related unemployment than population comparisons.^5^ Thus, there is a need to improve career guidance for childhood cancer survivors to help them select areas of work in which they can employ their competences and compensate potential physical or cognitive deficiencies related to their former cancer disease.

Our data showed an increased risk for low income for survivors in all diagnostic groups except for survivors of other and unspecified neoplasms. We also found that patients diagnosed with leukemia or a CNS tumor were at particular risk for having low income, which has also been shown in the Canadian longitudinal study^13^ and other previous studies, mainly using cross‐sectional income data.^9^, ^12^, ^26^ Cancer‐related cognitive deficits have been shown to be most common in survivors of CNS tumors or survivors of ALL.^27^ A systematic review and meta‐analysis from the Children's Oncology Group showed that CNS tumor survivors are at elevated risk for poor social attainment outcomes, such as being more likely to have completed compulsory education only and more likely to be unemployed compared to cancer‐free peers.^28^ Such underlying mechanism could offer an explanation for the observed lower income in our study. Leukemia patients in turn receive extended treatment (up to 2–2.5 years), which along with treatment‐related toxicity may require longer absence from school and social contacts in comparison with most other cancer types.

We found that survivors diagnosed at older age (16–19 years) were more likely to have middle/high income than those diagnosed at a younger age. Our results are in line with the Canadian longitudinal study where they found age at diagnosis to be a significant positive predictor of income.^13^ Also, in the Swiss Childhood Cancer Survivor Study survivors diagnosed from 15 to 20 years of age had higher income than survivors diagnosed at age of 0 to 5 years.^9^ In our study, some survivors diagnosed at 15 to 20 years of age may still undergo cancer treatment in the beginning of follow‐up which might interfere with their income situation. However, a similar pattern was observed in our sensitivity analysis including 5‐year survivors only. Younger age at cancer diagnosis is shown to be a risk factor for cancer‐related cognitive dysfunction, due to treatments coinciding with critical periods of brain development with the risk varying between cancer types.^27^

We found that the risk for low income was highest in the earlier diagnostic periods from 1971 to 1999 with the gap partially disappearing in the most recent diagnostic period from 2000 to 2009. Past and ongoing clinical efforts to develop less toxic cancer treatment regimens may provide an explanation and suggest a possible decrease in the treatment burden of some childhood cancers.^30^ One can also presume that better social support for patients and families may have also contributed to this development.

To consider the impact of childhood cancer on income in the low earning student population, we performed a separate analysis for groups studying and not studying. Despite students receiving benefits from the Nordic governments, our data showed that when studying, childhood cancer survivors are at increased risk of remaining in low income compared to their peers.

Childhood cancer survivors had overall 10% less transitions from low income to non‐low income, and 12% more transitions from non‐low income to low income than population comparisons. Childhood cancer diagnosis is a rare event, but in the childhood cancer survivor population of 500,000 in the European Union, these numbers are likely to be significant.^29^ However, the overall risk may also reflect earlier diagnostic periods with more toxic treatment and therefore it is likely to be reduced in the future. On the contrary, as the childhood cancer survival increases, the survivor population needing support is also growing.

Strengths of our study include nation‐wide registry data pooled from three Nordic countries, with similar welfare systems and population registries. The use of registry‐based information minimizes the risk of reporting or selection bias. Disposable income was retrieved from registries based on the information collected from the administration of taxes reducing the risk of information bias and ensuring virtually complete information. Our longitudinal income data offered us the opportunity to explore the effects of age and follow‐up time to the risk for low income. To account for the impact of parental socioeconomic status on income, we adjusted the analyses for highest parental education which, however, did not notably impact the results. With the sensitivity analyses including only 5‐years survivors of childhood cancer we controlled for the possible effect of ongoing cancer treatment among survivors diagnosed with cancer at older age.

Our study also had some limitations. We used the at‐risk‐of‐poverty threshold in our outcome definition, thus we do not report the absolute income differences. As a result, there are likely to be persons with somewhat similar income near the threshold but in different income categories. Separation of the source of income was not possible using the information available. Our main outcome (transition) is based on individual annual income over time in a matched setting.

In conclusion, our findings show higher risk of childhood cancer survivors to have a low income. The Children's Oncology Group releases “Long‐Term Follow‐Up Guidelines for Survivors of Childhood, Adolescent, and Young Adult Cancers”, where adverse psychosocial/quality of life effects are mentioned as potential late effects to be followed.^31^ Targeted supportive measures, such as career counseling and support and guidance in managing within the social security system may help to reduce these socioeconomic disparities. Vulnerable survivors may also need enhanced financial support and benefits. Follow‐up and support could be centralized in specialized late effect outpatient clinics with expertise on childhood cancer survivors and the possible somatic and socioeconomic challenges that can follow the survivors into adulthood.

## AUTHOR CONTRIBUTIONS

**Anniina Kyrönlahti:** Conceptualization (equal); data curation (equal); formal analysis (lead); investigation (lead); methodology (equal); writing – original draft (lead); writing – review and editing (lead). **Friederike Erdmann:** Conceptualization (equal); data curation (equal); investigation (equal); methodology (equal); writing – review and editing (equal). **Maria Feychting:** Conceptualization (equal); investigation (equal); methodology (equal); writing – review and editing (equal). **Line Elmerdahl Frederiksen:** Data curation (equal); investigation (equal); writing – review and editing (equal). **Elli Hirvonen:** Data curation (equal); formal analysis (supporting); writing – review and editing (supporting). **Liisa Maria Korhonen:** Investigation (equal); writing – review and editing (supporting). **Anja Krøyer:** Data curation (equal); writing – review and editing (equal). **Luzius Mader:** Conceptualization (equal); investigation (equal); writing – review and editing (equal). **Nea Malila:** Conceptualization (equal); investigation (equal); methodology (equal); writing – review and editing (equal). **Hanna Mogensen:** Conceptualization (equal); data curation (equal); investigation (equal); methodology (equal); writing – review and editing (equal). **Camilla Pedersen:** Investigation (equal); project administration (lead); writing – review and editing (equal). **Mats Talbäck:** Conceptualization (equal); data curation (equal); investigation (equal); methodology (equal); writing – review and editing (supporting). **Mervi Taskinen:** Conceptualization (equal); investigation (equal); methodology (equal); writing – review and editing (equal). **Jeanette Falck Winther:** Conceptualization (equal); data curation (equal); funding acquisition (lead); investigation (equal); methodology (equal); writing – review and editing (equal). **Laura Madanat‐Harjuoja:** Conceptualization (equal); investigation (equal); methodology (equal); writing – review and editing (equal). **Janne Pitkäniemi:** Conceptualization (equal); formal analysis (supporting); investigation (equal); methodology (equal); writing – review and editing (equal).

## CONFLICT OF INTEREST STATEMENT

The authors declare no competing interests. The funders had no role in study design, data collection/analysis/interpretation, or writing of the manuscript.

## ETHICS STATEMENT

The SALiCCS research programme was approved by the Regional Ethical Review Board in Stockholm, Sweden (2016/25–31/5, 2016/1561–32, 2017/1656–32, 2017/1990–32, 2017/2340–32, 2018/1165–32), Findata (THL/5543/14.06.00/2020) prolonging the former approvals by the National Institute for Health and Welfare and Social Insurance (KELA), and Statistics Finland (TK‐53‐394‐17). The SALiCCS research programme is listed in a local archive (2018‐DCRC‐0044) at the Danish Cancer Society Research Center, which replaces the former approval from the Danish Data Protection Agency. Pseudonymized individual‐level data were stored, linked, analyzed, and accessed remotely at a secure joint project platform at Statistics Denmark.

## Supporting information


Data S1:

## Data Availability

According to Danish, Finnish, and Swedish laws and regulations, individual‐level sensitive data can be made available only for researchers who fulfill legal requirements for access to personal sensitive data. Please contact the principal investigator Jeanette Falck Winther (jeanette@cancer.dk) for further questions about data access.

## References

[1] Gatta G , Botta L , Rossi S , et al. Childhood cancer survival in Europe 1999–2007: results of EUROCARE‐5—a population‐based study. Lancet Oncol. 2014;15:35‐47. 24314616 10.1016/S1470-2045(13)70548-5

[2] Allemani C , Matsuda T , Di Carlo V , et al. Global surveillance of trends in cancer survival 2000–14 (CONCORD‐3): analysis of individual records for 37 513 025 patients diagnosed with one of 18 cancers from 322 population‐based registries in 71 countries. Lancet. 2018;391:1023‐1075. 29395269 10.1016/S0140-6736(17)33326-3 PMC5879496

[3] Robison LL , Hudson MM . Survivors of childhood and adolescent cancer: life‐long risks and responsibilities. Nat Rev Cancer. 2014;14:61‐70. 24304873 10.1038/nrc3634 PMC6425479

[4] Frederiksen LE , Erdmann F , Mader L , et al. Psychiatric disorders in childhood cancer survivors in Denmark, Finland, and Sweden: a register‐based cohort study from the SALiCCS research programme. Lancet Psychiatry. 2022;9:35‐45. 34822758 10.1016/S2215-0366(21)00387-4

[5] Frederiksen LE , Pedersen C , Mogensen H , et al. Employment status and occupational positions of childhood cancer survivors from Denmark, Finland and Sweden: a Nordic register‐based cohort study from the SALiCCS research programme. Lancet Reg Health Eur. 2022;12:100258. 34901911 10.1016/j.lanepe.2021.100258 PMC8640515

[6] Frederiksen LE , Mader L , Feychting M , et al. Surviving childhood cancer: a systematic review of studies on risk and determinants of adverse socioeconomic outcomes. Int J Cancer. 2019;144:1796‐1823. 30098012 10.1002/ijc.31789

[7] Braveman P , Gottlieb L . The social determinants of health: it's time to consider the causes of the causes. Public Health Rep. 2014;129:19‐31. 10.1177/00333549141291S206 PMC3863696 24385661

[8] Kirchhoff AC , Krull KR , Ness KK , et al. Occupational outcomes of adult childhood cancer survivors: a report from the childhood cancer survivor study. Cancer. 2011;117:3033‐3044. 21246530 10.1002/cncr.25867 PMC3547616

[9] Wengenroth L , Sommer G , Schindler M , et al. Income in adult survivors of childhood cancer. PLoS One. 2016;11:e0155546. 27213682 10.1371/journal.pone.0155546 PMC4877063

[10] Ahomäki R , Harila‐Saari A , Matomäki J , Lähteenmäki PM . Non‐graduation after comprehensive school, and early retirement but not unemployment are prominent in childhood cancer survivors—a Finnish registry‐based study. J Cancer Surviv. 2017;11:284‐294. 27714627 10.1007/s11764-016-0574-z

[11] Gunnes MW , Lie RT , Bjørge T , et al. Economic independence in survivors of cancer diagnosed at a young age: a Norwegian national cohort study. Cancer. 2016;122:3873‐3882. 27518040 10.1002/cncr.30253 PMC5157778

[12] Boman KK , Lindblad F , Hjern A . Long‐term outcomes of childhood cancer survivors in Sweden: a population‐based study of education, employment, and income. Cancer. 2010;116:1385‐1391. 20087961 10.1002/cncr.24840

[13] Teckle P , Peacock S , McBride ML , Bentley C , Goddard K , Rogers P . Long‐term effects of cancer on earnings of childhood, adolescent and young adult cancer survivors – a population‐based study from British Columbia, Canada. BMC Health Serv Res. 2018;18:826. 30382843 10.1186/s12913-018-3617-5 PMC6211561

[14] Erdmann F , Frederiksen LE , Mogensen H , et al. Cohort profile: the socioeconomic consequences in adult life after childhood cancer in Scandinavia (SALiCCS) research Programme. Front Oncol. 2021;11:752948. 34900702 10.3389/fonc.2021.752948 PMC8662544

[15] Laugesen K , Ludvigsson JF , Schmidt M , et al. Nordic health registry‐based research: a review of health care systems and key registries. Clin Epidemiol. 2021;13:533‐554. 34321928 10.2147/CLEP.S314959 PMC8302231

[16] Thygesen LC , Daasnes C , Thaulow I , Brønnum‐Hansen H . Introduction to Danish (nationwide) registers on health and social issues: structure, access, legislation, and archiving. Scand J Public Health. 2011;39:12‐16. 21898916 10.1177/1403494811399956

[17] Official Statistics of Finland (OSF) . Total Statistics on Income Distribution. Statistics Finland; 2020. http://www.stat.fi/til/tjkt/index_en.html Accessed October 29, 2021.

[18] Official Statistics of Finland (OSF) . Students and Qualifications of Educational Institutions. Statistics Finland; 2020. http://www.stat.fi/til/opiskt/index_en.html Accessed October 29, 2021.

[19] Official Statistics of Finland (OSF) . Employment. Statistics Finland; 2020. http://www.stat.fi/til/tyokay/index_en.html Accessed October 29, 2021.

[20] Ludvigsson JF , Svedberg P , Olén O , Bruze G , Neovius M . The longitudinal integrated database for health insurance and labour market studies (LISA) and its use in medical research. Eur J Epidemiol. 2019;34:423‐437. 30929112 10.1007/s10654-019-00511-8 PMC6451717

[21] Eurostat. Income poverty statistics 2021. https://ec.europa.eu/eurostat/statistics‐explained/index.php?title=Glossary:At‐risk‐of‐poverty_rate Accessed October 6, 2021

[22] Danmarks Statistik . TIMES variabel, DISPON_NY. https://www.dst.dk/da/Statistik/dokumentation/Times/personindkomst/dispon‐ny (Accessed August 31, 2020)

[23] Steliarova‐Foucher E , Stiller C , Lacour B , Kaatsch P . International classification of childhood cancer, third edition. Cancer. 2005;103:1457‐1467. 15712273 10.1002/cncr.20910

[24] Whalen T , Boeri M . Measuring discontinuity in binary longitudinal data: applications to drug use trajectories. Sociol Methods Res. 2014;43:248‐279. 25309006 10.1177/0049124113511594 PMC4190590

[25] Benjamini Y , Hochberg Y . Controlling the false discovery rate: a practical and powerful approach to multiple testing. J R Stat Soc Ser B Methodol. 1995;57:289‐300.

[26] Kirchhoff AC , Krull KR , Ness KK , et al. Occupational outcomes of adult childhood cancer survivors. Cancer. 2011;117:3033‐3044. 21246530 10.1002/cncr.25867 PMC3547616

[27] Castellino SM , Ullrich NJ , Whelen MJ , Lange BJ . Developing interventions for cancer‐related cognitive dysfunction in childhood cancer survivors. J Natl Cancer Inst. 2014;106:186. 10.1093/jnci/dju186 PMC4155432 25080574

[28] Schulte F , Kunin‐Batson AS , Olson‐Bullis BA , et al. Social attainment in survivors of pediatric central nervous system tumors: a systematic review and meta‐analysis from the Children's Oncology Group. J Cancer Surviv. 2019;13:921‐931. 31625086 10.1007/s11764-019-00808-3 PMC6900341

[29] Vassal G , Schrappe M , Pritchard‐Jones K , et al. The SIOPE strategic plan: a European cancer plan for children and adolescents. J Cancer Policy. 2016;8:17‐32.

[30] Bartram J , Veys P , Vora A . Improvements in outcome of childhood acute lymphoblastic leukaemia (ALL) in the UK–a success story of modern medicine through successive UKALL trials and international collaboration. Br J Haematol. 2020;191:562‐567. 33190256 10.1111/bjh.17162

[31] Children's Oncology Group . Long‐Term Follow‐up Guidelines for Survivors of Childhood, Adolescent, and Young Adult Cancers, Version 5.0. Children's Oncology Group; 2018. 10.1001/jamaoncol.2024.6812 PMC12188901 39976936

