# Supplementary material for: Income disparities between adult childhood cancer survivors and their peers—A register‐based cohort study from the SALiCCS research programme
Source: Cancer Med. 2023 Jun 12;12(15):16455–68. doi: 10.1002/cam4.6218 (PMC10469706; doi:10.1002/cam4.6218)
Supplement: Supplementary file 1 — Data S1: [file CAM4-12-16455-s001.docx]

**Income disparities between adult childhood cancer survivors and their peers** – **a register-based cohort study from the SALiCCS research programme**

**Supplementary Material**

**Figure S1.** Flow chart of the inclusion and exclusion criteria of childhood cancer survivors and population comparisons.**2**

**Table S1**. Number of individuals in different states of income among childhood cancer survivors (diagnosed with cancer at the age of 0 to 19 years from 1971 to 2009)1 and their population comparisons (matched by birth year, sex, and country) by initial state of income and diagnostic group (by ICCC).**3**

**Table S2.** Adjusted RRs with 95 % CIs for the number of individuals in low income^1^ on an annual basis for childhood cancer survivors (diagnosed with cancer at the age of 0 to 19 years between 1971 and 2009) compared to population comparisons (matched by birth year and sex) by country.**4**

**Table S3**. Characteristics and adjusted RRs with 95 % CIs for number of transitions from low income to middle/high income^1^ for childhood cancer survivors^2^ compared to population comparisons (matched by birth year and sex) by country.**5**

**Table S4.** Characteristics and adjusted RRs with 95 % CIs for number of observations in transitions from middle/high income to low income^1^ for childhood cancer survivors^2^ compared to population comparisons (matched by birth year and sex) by country.**6**

**Table S5.** Adjusted RRs with 95 % CIs for number of observations in transitions from low income to middle/high income^1^, and from middle/high to low income for 5-year survivors of childhood cancer^2^ compared to population comparisons (matched by birth year, sex, and country).**7**

**Table S6.** Number of individuals in different states of income among 5-year survivors of childhood cancer (diagnosed with cancer at the age of 0 to 19 years from 1971 to 2009)^1^ and their population comparisons (matched by birth year, sex, and country) by initial income category.**9**

**Table S7.** Number of individuals in different states of income among childhood cancer survivors (diagnosed with cancer at the age of 0 to 19 years from 1971 to 2009)^1^ and their population comparisons (matched by birth year, sex, and country) at ages 30-40 years by initial state of income.**10**

**Figure S1. Flow chart of the inclusion and exclusion criteria of childhood cancer survivors and population comparisons.**

**
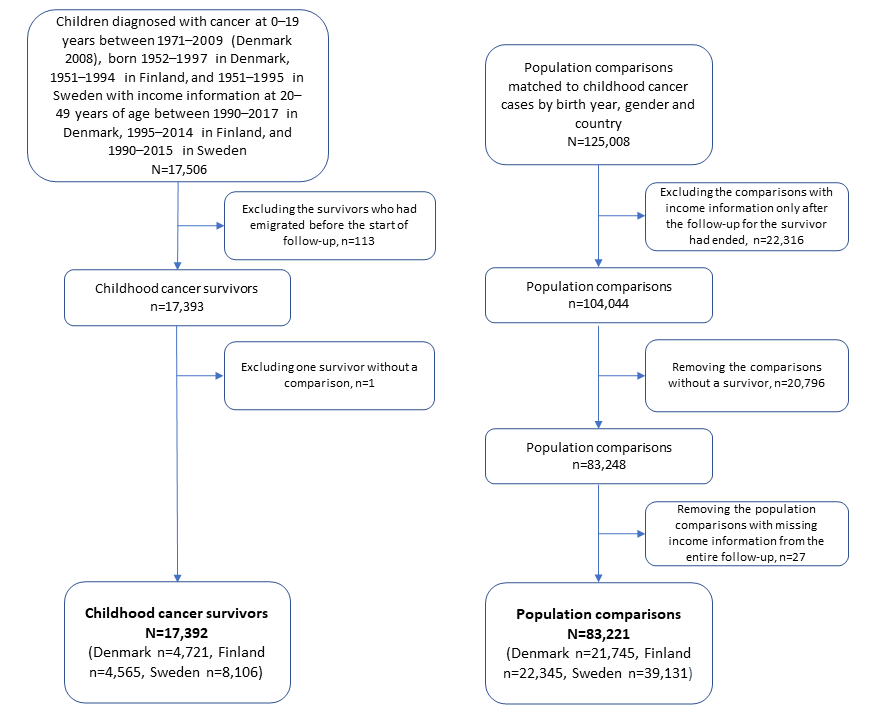
**

| **Table S1. Number of individuals in different states of income among childhood cancer survivors (diagnosed with cancer at the age of 0 to 19 years from 1971 to 2009)1 and their population comparisons (matched by birth year, sex, and country) by initial state of income and diagnostic group (by ICCC).** | | | | | | | | | | | | | | | | | | | | | | | | | | | |  |  |  |  |  |  |
| --- | --- | --- | --- | --- | --- | --- | --- | --- | --- | --- | --- | --- | --- | --- | --- | --- | --- | --- | --- | --- | --- | --- | --- | --- | --- | --- | --- | --- | --- | --- | --- | --- | --- |
|  | **Leukemias** | | | | | **Lymphomas** | | | | | | | | | **CNS tumors^3^** | | | | | | | | | | **Other solid tumors^4^** | | | | | | | | |
|  | **Survivors** | **Population  comparisons** | |  | | **Survivors** | | | **Population  comparisons** | | |  | | | **Survivors** | | | **Population  comparisons** | | | |  | | **Survivors** | | | **Population  comparisons** | | | |  | | |
|  | N (%) | N (%) | | p-value^2^ | | N (%) | | | N (%) | | | p-value^2^ | | | N (%) | | | N (%) | | | | p-value^2^ | | N (%) | | | N (%) | | | | p-value^2^ | | |
| **Initial category low income** | **739 (23.3)** | **3,342 (22.0)** | |  | | **605 (22.3)** | | | **2,847 (21.7)** | | |  | | | **873 (21.5)** | | | **4,113 (20.9)** | | | |  | | **1,291 (19.7)** | | | **6,345 (20.1)** | | | |  | | |
| Remaining in low income during the entire follow-up | 109 (3.4) | 482 (3.2) | | 0.89 | | 68 (2.5) | | | 344 (2.6) | | | 0.70 | | | 107 (2.6) | | | 547 (2.8) | | | | 0.54 | | 177 (2.7) | | | 863 (2.7) | | | | 0.95 | | |
| Permanent transition to middle/high income | 274 (8.7) | 1,508 (9.9) | | <0.01 | | 227 (8.4) | | | 1,199 (9.1) | | | 0.066 | | | 367 (9.0) | | | 1,783 (9.1) | | | | 0.54 | | 512 (7.8) | | | 2,670 (8.4) | | | | 0.18 | | |
| Transition to middle/high income and permanently back to low income | 88 (2.8) | 385 (2.5) | | 0.89 | | 090 (3.3) | | | 323 (2.5) | | | 0.038 | | | 130 (3.2) | | | 469 (2.4) | | | | <0.01 | | 164 (2.5) | | | 686 (2.2) | | | | 0.11 | | |
| Multiple transitions | 268 (8.5) | 967 (6.4) | | <0.01 | | 220 (8.1) | | | 981 (7.5) | | | 0.53 | | | 269 (6.6) | | | 1,314 (6.7) | | | | 0.54 | | 438 (6.7) | | | 2,126 (6.7) | | | | 0.91 | | |
| **Initial category middle/high income** | **2,426 (76.7)** | **11,847 (78.0)** | |  | | **2,107 (77.7)** | | | **10,279 (78.3)** | | |  | | | **3,188 (78.5)** | | | **15,537 (79.1)** | | | |  | | **5,266 (80.3)** | | | **25,257 (79.9)** | | | |  | | |
| Remaining in middle/high income during the entire follow-up | 1,316 (41.6) | 6,960 (45.8) | | <0.01 | | 1,123 (41.4) | | | 5,925 (45.1) | | | <0.01 | | | 1,579 (38.9) | | | 9,085 (46.2) | | | | <0.01 | | 2,808 (42.8) | | | 14,513 (45.9) | | | | <0.01 | | |
| Permanent transition to low income | 298 (9.4) | 1,052 (6.9) | | <0.01 | | 197 (7.3) | | | 801 (6.1) | | | 0.038 | | | 513 (12.6) | | | 1,290 (6.6) | | | | <0.01 | | 500 (7.6) | | | 1,911 (6.0) | | | | <0.01 | | |
| Transition to low income and permanently back to middle/high income | 432 (13.6) | 2,127 (14.0) | | 0.89 | | 390 (14.4) | | | 1,886 (14.4) | | | 0.89 | | | 516 (12.7) | | | 2,826 (14.4) | | | | 0.012 | | 1,008 (15.4) | | | 4,720 (14.9) | | | | 0.61 | | |
| Multiple transitions | 380 (12.0) | 1,708 (11.2) | | 0.19 | | 397 (14.6) | | | 1,667 (12.7) | | | 0.014 | | | 580 (14.3) | | | 2,336 (11.9) | | | | <0.01 | | 950 (14.5) | | | 4,113 (13.0) | | | | <0.01 | | |
| ^1^From 1971 to 2008 in Denmark. | | | | |  | | |  | | | | |  |  | | |  | | | |  | |  | | | |  | | |  | |  |  |
| ^2^Corrected for multiple comparisons using Benjamini &Hochberg. | | | | | | | | | | | | | | | | |  | | | |  | |  | | | |  | | |  | |  |  |
| ^3^Central nervous system tumors. | | | | |  | | |  | | | | |  |  | | |  | | | |  | |  | | | |  | | |  | |  |  |
| ^4^Neuroblastomas, retinoblastomas, renal tumors, hepatic tumors, bone tumors, soft tissue sarcomas, germ-cell tumors, carcinomas, and other and unspecified neoplasms. | | | | | | | | | | | | | | | | | | | | | | | | | | | | | | | | |  |
|  | | | | | | | | | | | | | | | | | | | | | | | | | | | | | | | | |  |
| **Table S2. Adjusted RRs with 95 % CIs for the number of individuals in low income^1^ on an annual basis for childhood cancer survivors (diagnosed with cancer at the age of 0 to 19 years between 1971 and 2009) compared to population comparisons (matched by birth year and sex) by country.** | | | | | | | | | | | | | | | | | | | | | | | | | | | |  |  |  |  |  |  |
|  | | | **Denmark** | | | | | | | **Finland** | | | | | | | | | **Sweden** | | | | | | | | |  |  |  |  |  |  |
|  | | | Adjusted^2^ RR (95 % CI) | | | | Adjusted^3^ RR (95 % CI) | | | | Adjusted^2^ RR (95 % CI) | | | | | Adjusted^3^ RR (95 % CI) | | | | Adjusted^2^ RR (95 % CI) | | | | | | Adjusted^3^ RR (95 % CI) | | |  |  |  |  |  |
| **During the entire study period** | | |  | | | |  | | | |  | | | | |  | | | |  | | | | | |  | | |  |  |  |  |  |
| Low income | | | 1.08 (1.06-1.11) | | | | 1.10 (1.08-1.13) | | | | 1.20 (1.18-1.22) | | | | | 1.13 (1.11-1.16) | | | | 1.20 (1.18-1.21) | | | | | | 1.19 (1.17-1.21) | | |  |  |  |  |  |
| **By the age of 35** | | |  | | | |  | | | |  | | | | |  | | | |  | | | | | |  | | |  |  |  |  |  |
| Low income | | | 1.08 (1.06-1.11) | | | | 1.10 (1.08-1.13) | | | | 1.20 (1.18-1.22) | | | | | 1.13 (1.11-1.16) | | | | 1.20 (1.18-1.21) | | | | | | 1.19 (1.17-1.21) | | |  |  |  |  |  |
| ^1^Annual disposable income at ages 20 to 50 years was retrieved between 1990 and 2017 for Denmark, 1990 and 2015 for Sweden, and 1995 and 2014 for  Finland, and dichotomized to low income and middle/high income based on the at-risk-of-poverty threshold defined by Eurostat. | | | | | | | | | | | | | | | | | | | | | | | | | | | |  |  |  |  |  |  |
| ^2^Adjusted for attained age during follow-up, calendar period, and sex. | | | | | | | | | | | | | | | | | | | | | | | | | | | |  |  |  |  |  |  |
| ^3^Adjusted for attained age during follow-up, calendar period, sex, and highest parental education. | | | | | | | | | | | | | | | | | | | | | | | | | | | |  |  |  |  |  |  |

| **Table S3. Characteristics and adjusted RRs with 95 % CIs for number of transitions from low income to middle/high income^1^ for childhood cancer survivors^2^ compared to population comparisons (matched by birth year and sex) by country.** | | | | | | |
| --- | --- | --- | --- | --- | --- | --- |
|  | **Survivors** | | **Population comparisons** | |  |  |
|  | Number of individuals remaining in low income (%)^3^ | Number of transitions to middle/high income (%)^3^ | Number of individuals remaining in low income (%)^3^ | Number of transitions to middle/high income (%)^3^ | Adjusted^4^ RR (95 % CI) | Adjusted^5^ RR (95 % CI) |
| **Country** |  |  |  |  |  |  |
| Denmark | 5,725 (65.3) | 3,047 (34.7) | 22,568 (64.1) | 12,666 (35.9) | 0.97 (0.94-1.00) | 0.96 (0.93-0.99) |
| Finland | 8,199 (72.8) | 3,068 (27.2) | 30,335 (66.5) | 15,300 (33.5) | 0.86 (0.83-0.88) | 0.90 (0.87-0.94) |
| Sweden | 11,674 (68.4) | 5,388 (31.6) | 43,666 (63.7) | 24,853 (36.3) | 0.90 (0.88-0.93) | 0.90 (0.88-0.93) |
| ^1^Annual disposable income at ages 20 to 50 years was retrieved between 1990 and 2017 for Denmark, 1990 and 2015 for Sweden, and 1995 and 2014 for Finland, and dichotomized to low income and middle/high income based on the at-risk-of-poverty threshold defined by Eurostat. | | | | | | |
| ^2^Diagnosed with cancer at the age of 0 to 19 years from 1971 to 2009 for Finland and Sweden, and from 1971 to 2008 for Denmark. | | | | | | |
| ^3^Total number of observations during the entire study period. | | | | | |  |
| ^4^Adjusted for attained age during follow-up, calendar period, and sex. | | | | | |  |
| ^5^Adjusted for attained age during follow-up, calendar period, sex, and highest parental education. | | | | | |  |

| **Table S4. Characteristics and adjusted RRs with 95 % CIs for number of observations in transitions from middle/high income to low income^1^ for childhood cancer survivors^2^ compared to population comparisons (matched by birth year and sex) by country.** | | | | | | |
| --- | --- | --- | --- | --- | --- | --- |
|  | **Survivors** | | **Population comparisons** | |  |  |
|  | Number of individuals remaining in middle/high income (%)^3^ | Number of transitions to low income (%)^3^ | Number of individuals remaining in middle/high income (%)^3^ | Number of transitions to low income (%)^3^ | Adjusted^4^ RR (95 % CI) | Adjusted^5^ RR (95 % CI) |
| **Country** |  |  |  |  |  |  |
| Denmark | 50,347 (93.9) | 3,256 (6.1) | 215,183 (94.3) | 13,126 (5.7) | 1.07 (1.03-1.11) | 1.08 (1.04-1.12) |
| Finland | 35,785 (92.0) | 3,132 (8.0) | 183,279 (92.9) | 14,025 (7.1) | 1.13 (1.09-1.17) | 1.12 (1.07-1.17) |
| Sweden | 75,985 (93.2) | 5,552 (6.8) | 374,149 (94.1) | 23,625 (5.9) | 1.16 (1.12-1.19) | 1.15 (1.12-1.18) |
| ^1^Annual disposable income at ages 20 to 50 years was retrieved between 1990 and 2017 for Denmark, 1990 and 2015 for Sweden, and 1995 and 2014 for Finland, and dichotomized to low income and middle/high income based on the at-risk-of-poverty threshold defined by Eurostat. | | | | | | |
| ^2^Diagnosed with cancer at the age of 0-19 years from 1971 to 2009 for Finland and Sweden, and from 1971 to 2008 for Denmark. | | | | | | |
| ^3^Total number of observations during the entire study period. | | | | | |  |
| ^4^Adjusted for age during follow-up, calendar period, and sex. | | | | | |  |
| ^5^Adjusted for age during follow-up, calendar period, sex, and highest parental education. | | | | | |  |

| **Table S5. Adjusted RRs with 95 % CIs for number of observations in transitions from low income to middle/high income^1^, and from middle/high to low income for 5-year survivors of childhood cancer^2^ compared to population comparisons (matched by birth year, sex, and country).** | | | | | | | |  |  |  |  |
| --- | --- | --- | --- | --- | --- | --- | --- | --- | --- | --- | --- |
|  | **Transitions from low to  middle/high income** | | | **Transitions from middle/high to  low income** | | | |  |  |  |  |
|  | Adjusted^3^ RR (95 % CI) | Adjusted^4^ RR (95 % CI) | | Adjusted^3^ RR (95 % CI) | | Adjusted^4^ RR (95 % CI) | |  |  |  |  |
| **Total** | 0.90 (0.88-0.91) | 0.91 (0.90-0.93) | | 1.12 (1.10-1.15) | | 1.12 (1.10-1.14) | |  |  |  |  |
| **Sex** |  |  | |  | |  | |  |  |  |  |
| Male | 0.92 (0.90-0.95) | 0.94 (0.92-0.97) | | 1.13 (1.10-1.16) | | 1.13 (1.09-1.16) | |  |  |  |  |
| Female | 0.93 (0.91-0.95) | 0.95 (0.92-0.97) | | 1.12 (1.09-1.15) | | 1.11 (1.08-1.15) | |  |  |  |  |
| **Follow-up period** |  |  | |  | |  | |  |  |  |  |
| 1990-1994 | 0.97 (0.91-1.05) | 0.97 (0.90-1.05) | | 0.99 (0.90-1.08) | | 0.96 (0.87-1.06) | |  |  |  |  |
| 1995-1999 | 0.97 (0.92-1.01) | 0.96 (0.92-1.01) | | 1.04 (0.98-1.10) | | 1.05 (0.99-1.12) | |  |  |  |  |
| 2000-2004 | 0.94 (0.91-0.98) | 0.96 (0.93-1.00) | | 1.13 (1.08-1.19) | | 1.11 (1.06-1.17) | |  |  |  |  |
| 2005-2009 | 0.89 (0.86-0.92) | 0.92 (0.88-0.95) | | 1.17 (1.12-1.21) | | 1.17 (1.12-1.22) | |  |  |  |  |
| 2010-2014 | 0.84 (0.81-0.86) | 0.86 (0.83-0.88) | | 1.15 (1.11-1.19) | | 1.15 (1.10-1.19) | |  |  |  |  |
| 2015-2017 | 0.90 (0.82-0.98) | 0.89 (0.82-0.97) | | 1.09 (0.99-1.19) | | 1.09 (0.99-1.20) | |  |  |  |  |
| **Age during follow-up** |  |  | |  | |  | |  |  |  |  |
| 20-24 years | 0.95 (0.92-0.98) | 0.95 (0.92-0.98) | | 1.05 (1.01-1.09) | | 1.05 (1.01-1.09) | |  |  |  |  |
| 25-29 years | 0.94 (0.91-0.97) | 0.95 (0.92-0.98) | | 1.12 (1.08-1.16) | | 1.10 (1.05-1.14) | |  |  |  |  |
| 30-34 years | 0.86 (0.82-0.89) | 0.88 (0.84-0.92) | | 1.17 (1.11-1.22) | | 1.16 (1.10-1.22) | |  |  |  |  |
| 35-39 years | 0.81 (0.76-0.85) | 0.82 (0.77-0.87) | | 1.20 (1.13-1.28) | | 1.21 (1.14-1.29) | |  |  |  |  |
| 40-44 years | 0.74 (0.69-0.80) | 0.74 (0.68-0.81) | | 1.19 (1.10-1.29) | | 1.23 (1.13-1.34) | |  |  |  |  |
| 45-49 years | 0.81 (0.73-0.90) | 0.86 (0.77-0.97) | | 1.16 (1.05-1.29) | | 1.28 (1.14-1.44) | |  |  |  |  |
| **Cancer type** |  |  | |  | |  | |  |  |  |  |
| Leukemias | 0.89 (0.85-0.92) | 0.90 (0.86-0.94) | | 1.13 (1.08-1.18) | | 1.10 (1.05-1.16) | |  |  |  |  |
| Lymphomas | 0.98 (0.94-1.02) | 0.99 (0.95-1.03) | | 1.14 (1.08-1.20) | | 1.14 (1.08-1.20) | |  |  |  |  |
| CNS tumors^5^ | 0.80 (0.77-0.83) | 0.82 (0.78-0.85) | | 1.22 (1.17-1.26) | | 1.19 (1.14-1.25) | |  |  |  |  |
| Neuroblastomas | 0.81 (0.71-0.92) | 0.87 (0.76-1.01) | | 1.09 (0.93-1.27) | | 1.06 (0.89-1.27) | |  |  |  |  |
| Retinoblastomas | 0.86 (0.77-0.96) | 0.84 (0.74-0.96) | | 1.06 (0.93-1.22) | | 1.05 (0.90-1.22) | |  |  |  |  |
| Renal tumors | 1.01 (0.93-1.09) | 1.01 (0.93-1.10) | | 1.12 (1.02-1.24) | | 1.11 (0.99-1.24) | |  |  |  |  |
| Hepatic tumors | 0.84 (0.63-1.13) | 0.85 (0.60-1.21) | | 1.11 (0.79-1.58) | | 1.01 (0.68-1.52) | |  |  |  |  |
| Bone tumors | 0.91 (0.83-1.00) | 0.95 (0.87-1.05) | | 1.07 (0.97-1.19) | | 1.12 (1.00-1.25) | |  |  |  |  |
| Soft tissue sarcomas | 0.88 (0.81-0.96) | 0.92 (0.84-1.00) | | 1.06 (0.97-1.16) | | 1.12 (1.01-1.24) | |  |  |  |  |
| Germ-cell neoplasms | 0.94 (0.88-1.00) | 0.94 (0.87-1.00) | | 1.05 (0.97-1.14) | | 1.07 (0.99-1.16) | |  |  |  |  |
| Carcinomas | 1.00 (0.95-1.05) | 1.01 (0.96-1.07) | | 1.07 (1.01-1.13) | | 1.06 (0.99-1.12) | |  |  |  |  |
| Other and unspecified neoplasms | 0.96 (0.86-1.07) | 1.04 (0.93-1.17) | | 0.99 (0.87-1.12) | | 1.04 (0.90-1.19) | |  |  |  |  |
| **Age at diagnosis** |  |  | |  | |  | |  |  |  |  |
| 0-4 years | 0.87 (0.83-0.90) | 0.88 (0.85-0.92) | | 1.17 (1.12-1.22) | | 1.14 (1.08-1.19) | |  |  |  |  |
| 5-9 years | 0.85 (0.82-0.89) | 0.88 (0.84-0.92) | | 1.07 (1.02-1.12) | | 1.07 (1.01-1.12) | |  |  |  |  |
| 10-15 years | 0.89 (0.86-0.92) | 0.91 (0.88-0.94) | | 1.13 (1.09-1.17) | | 1.13 (1.09-1.17) | |  |  |  |  |
| 16-19 years | 0.97 (0.94-1.00) | 0.97 (0.94-1.01) | | 1.12 (1.08-1.16) | | 1.13 (1.08-1.17) | |  |  |  |  |
| **Diagnostic period** |  |  | |  | |  | |  |  |  |  |
| 1971-1979 | 0.81 (0.78-0.85) | 0.83 (0.79-0.87) | | 1.12 (1.07-1.17) | | 1.12 (1.06-1.18) | |  |  |  |  |
| 1980-1989 | 0.89 (0.87-0.92) | 0.93 (0.90-0.97) | | 1.16 (1.12-1.20) | | 1.16 (1.12-1.20) | |  |  |  |  |
| 1990-1999 | 0.93 (0.90-0.95) | 0.93 (0.90-0.96) | | 1.11 (1.07-1.15) | | 1.11 (1.07-1.15) | |  |  |  |  |
| 2000-2009 | 0.94 (0.89-0.99) | 0.93 (0.88-0.98) | | 1.02 (0.95-1.09) | | 1.01 (0.95-1.08) | |  |  |  |  |
| **Studying** |  |  | |  | |  | |  |  |  |  |
| Total | 0.93 (0.90-0.97) | 0.95 (0.91-0.99) | | 1.01 (0.97-1.04) | | 1.01 (0.98-1.05) | |  |  |  |  |
| **Sex** |  |  | |  | |  | |  |  |  |  |
| Male | 0.97 (0.91-1.02) | 0.97 (0.91-1.03) | | 1.02 (0.97-1.07) | | 1.04 (0.99-1.09) | |  |  |  |  |
| Female | 0.91 (0.86-0.96) | 0.93 (0.88-0.98) | | 0.99 (0.95-1.04) | | 0.99 (0.94-1.04) | |  |  |  |  |
| **Not studying** |  |  | |  | |  | |  |  |  |  |
| Total | 0.88 (0.87-0.90) | 0.90 (0.88-0.92) | | 1.16 (1.14-1.19) | | 1.16 (1.13-1.19) | |  |  |  |  |
| **Sex** |  |  | |  | |  | |  |  |  |  |
| Male | 0.91 (0.89-0.94) | 0.94 (0.91-0.96) | | 1.16 (1.12-1.20) | | 1.16 (1.11-1.20) | |  |  |  |  |
| Female | 0.86 (0.83-0.88) | 0.87 (0.84-0.89) | | 1.17 (1.13-1.21) | | 1.16 (1.12-1.21) | |  |  |  |  |
| ^1^Annual disposable income at ages 20-50 years was retrieved between 1990 and 2017 for Denmark, 1990 and 2015 for Sweden, and 1995 and 2014 for Finland, and dichotomized to low income and middle/high income based on the at-risk-of-poverty threshold defined by Eurostat. | | | | | | | |  |  |  |  |
| ^2^Diagnosed with cancer at the age of 0-19 years from 1971 to 2009 for Finland and Sweden, and  from 1971 to 2008 for Denmark. | | | | | | | |  |  |  |  |
| ^3^Adjusted for age during follow-up, calendar period, sex, and country. | | | | | | | |  |  |  |  |
| ^4^Adjusted for age during follow-up, calendar period, sex, country, and highest parental education. | | | | | | | |  |  |  |  |
| ^5^Central nervous system tumors. | | | | | | | |  |  |  |  |
| **Table S6. Number of individuals in different states of income among 5-year survivors of childhood cancer (diagnosed with cancer at the age of 0 to 19 years from 1971 to 2009)^1^ and their population comparisons (matched by birth year, sex, and country) by initial income category.** | | | | | | | | | | | |
|  | | | **Survivors** | | **Population  comparisons** | |  | |  | |  |
|  | | | N (%) | | N (%) | | Adjusted^2^ RR (95 % CI) | | Adjusted^3^ RR (95 % CI) | |  |
| **Initial category low income** | | | **3,371 (20.9)** | | **15,256 (19.8)** | |  | |  | |  |
| Remaining in low income during the entire follow-up | | | 515 (3.2) | | 2,267 (2.9) | | 1.04 (0.96-1.14) | | 1.06 (0.96-1.16) | |  |
| Permanent transition to middle/high income | | | 1,325 (8.2) | | 6,538 (8.5) | | 0.93 (0.88-0.97) | | 0.93 (0.89-0.98) | |  |
| Transition to middle/high income and permanently back to low income | | | 445 (2.8) | | 1,759 (2.3) | | 1.13 (1.03-1.25) | | 1.15 (1.03-1.27) | |  |
| Multiple transitions | | | 1,086 (6.7) | | 4,692 (6.1) | | 1.04 (0.98-1.09) | | 1.02 (0.96-1.08) | |  |
| **Initial category middle/high income** | | | **12,765 (79.1)** | | **61,879 (80.2)** | |  | |  | |  |
| Remaining in middle/high income during the entire follow-up | | | 7,015 (43.5) | | 37,077 (48.1) | | 0.92 (0.90-0.93) | | 0.92 (0.91-0.94) | |  |
| Permanent transition to low income | | | 1,458 (9.0) | | 4,996 (6.5) | | 1.41 (1.34-1.49) | | 1.38 (1.30-1.47) | |  |
| Transition to low income and permanently back to middle/high income | | | 2,236 (13.9) | | 10,943 (14.2) | | 0.99 (0.95-1.03) | | 0.99 (0.95-1.04) | |  |
| Multiple transitions | | | 2,056 (12.7) | | 8,863 (11.5) | | 1.13 (1.08-1.18) | | 1.11 (1.06-1.17) | |  |
| ^1^From 1971 to 2008 in Denmark. | | | | | | | | | |  | |
| ^2^Adjusted for sex and country. | | | | | | | | | |  | |
| ^3^Adjusted for sex, country, and highest parental education. | | | | | | | | | |  | |

| **Table S7. Number of individuals in different states of income among childhood cancer survivors (diagnosed with cancer at the age of 0 to 19 years from 1971 to 2009)^1^ and their population comparisons (matched by birth year, sex, and country) at ages 30 to 40 years by initial income category.** | | | | |  |
| --- | --- | --- | --- | --- | --- |
|  | **Survivors** | **Population  comparisons** |  |  | |
|  | N (%) | N (%) | Adjusted^2^ RR (95 % CI) | Adjusted^3^ RR (95 % CI) | |
| **Initial category low income** | **1,579 (16.0)** | **6,172 (13.7)** |  |  | |
| Remaining in low income during the entire follow-up | 603 (6.1) | 2,008 (4.5) | 1.17 (1.09-1.26) | 1.19 (1.09-1.29) | |
| Permanent transition to middle/high income | 532 (5.4) | 2,424 (5.4) | 0.86 (0.80-0.93) | 0.85 (0.78-0.93) | |
| Transition to middle/high income and permanently back to low income | 209 (2.1) | 709 (1.6) | 1.15 (1.00-1.33) | 1.16 (0.99-1.37) | |
| Multiple transitions | 235 (2.4) | 1,031 (2.3) | 0.90 (0.79-1.02) | 0.91 (0.79-1.05) | |
| **Initial category middle/high income** | **8,270 (84.0)** | **38,880 (86.3)** |  |  | |
| Remaining in middle/high income during the entire follow-up | 6,260 (63.6) | 30,784 (68.3) | 0.96 (0.94-0.97) | 0.96 (0.94-0.97) | |
| Permanent transition to low income | 784 (8.0) | 2,490 (5.5) | 1.47 (1.37-1.60) | 1.47 (1.35-1.60) | |
| Transition to low income and permanently back to middle/high income | 770 (7.8) | 3,794 (8.4) | 0.96 (0.89-1.03) | 0.97 (0.89-1.05) | |
| Multiple transitions | 456 (4.6) | 1,812 (4.0) | 1.19 (1.07-1.31) | 1.21 (1.08-1.35) | |
| ^1^From 1971 to 2008 in Denmark. | | | |  | |
| ^2^Adjusted for sex and country. | | | |  | |
| ^3^Adjusted for sex, country, and highest parental education. | | | |  | |
